# Hydropower development and malaria transmission: A geospatial econometric study

**DOI:** 10.4102/jphia.v16i1.1397

**Published:** 2025-10-06

**Authors:** Callum J. Thomas

**Affiliations:** 1 Department of International Relations, Peking University, Beijing, China; 2 Department of Finance for Sustainable Development, Organisation for Economic Cooperation and Development, Paris, France

**Keywords:** difference-in-differences, regression modelling, geographic information systems, GIS, malaria incidence, malaria prevalence, hydrodams

## Abstract

**Background:**

In Western Africa, the causal relationship between hydropower project implementation and malaria transmission, remains understudied.

**Aim:**

This study assesses whether a causal correlation exists between hydropower development and malaria transmission outcomes across locally affected communities, using malaria incidence and prevalence as key indicators. Malaria incidence is measured as the number of clinical *Plasmodium falciparum* cases per person, while prevalence is the parasite rate of *P. falciparum* in children aged 2–10 years. The analysis focuses on *P. falciparum* given its severity across West Africa, along with the availability of consistent geospatial data.

**Setting:**

The study was conducted in Ghana, Côte d’Ivoire, and Gabon.

**Methods:**

Utilising multivariate Difference-in-Differences (DiD) regression models and geospatial analysis across pre- and post-dam periods, this study evaluates malaria outcomes within 15 km of hydropower sites.

**Results:**

The DiD estimator (Treatment_Post variable) suggests no statistically significant increase in malaria transmission following hydropower project implementation. Estimated effects are insignificant in Côte d’Ivoire (incidence: *p* = 0.210, prevalence: *p* = 0.200), Gabon (incidence: *p* = 0.990, prevalence: *p* = 0.990), and Ghana (incidence: *p* = 0.089, prevalence: *p* = 0.102), indicating no strong causal link at the 5% level. By contrast, environmental and socio-economic variables such as urbanisation, elevation, and climate factors consistently showed strong associations with malaria transmission (*p* < 0.01).

**Conclusion:**

Hydropower presence alone is not a primary driver of malaria dynamics.

**Contribution:**

This study provides the first large-scale geospatial analysis of malaria trends across hydropower projects in Western Africa, challenging traditional assumptions of a direct causal link and highlighting the need for interventions shaped by environmental and socio-economic factors.

## Introduction

The absence of electricity access in sub-Saharan Africa (SSA) represents a significant obstacle to the region’s economic progress. Insufficient power generation capacity, transmission and distribution networks and established utility systems create considerable hurdles for SSA’s socio-economic advancement. This is not only because of the fact that the world’s current energy mix and reliance on fossil fuels contribute to 40% of global CO_2_ emissions,^1^ but also because any economic future is predicated on a consistent provision of sustainable, affordable and universally accessible energy.

Currently, Chinese public development banks play a key role in financing Africa’s renewable energy transition, aligning with the global push to limit carbon emissions, improve energy grid efficiency and promote sustainable economic development.^2^ Sinohydro, as the world’s largest dam builder, has and will continue to play a pivotal role in building up the African continent’s hydroelectric energy infrastructure.^3^ While these projects contribute to increases in energy access and economic growth, they also introduce complex socio-environmental trade-offs. In recent year, growing criticisms have emerged around hydropower project implementation, highlighting not only environmental risks such as the loss of vital wetland areas,^4^ increased pollution and communicable diseases^5^ and biodiversity loss,^6^ but also socio-economic issues including the displacement of communities^7^ and inadequate environmental safeguards.^8^ However, there exists a research gap in assessing the direct malaria transmission impacts of hydrodam implementation, particularly in how different development players shape outcomes. While existing studies focus on environmental and social effects, they often overlook direct public health consequences. Studies, however, have established the fact that water reservoirs created by hydrodams provide ideal breeding grounds for Anopheles mosquitoes, the primary carriers of malaria.^9,10,11^ These stagnant water bodies, which often lack natural mosquito predators such as fish or tadpoles, extend the malaria transmission season beyond traditional rainy periods, exacerbating the disease burden in affected regions.^12,13^

The correlation between hydrodam construction and malaria transmission is not merely theoretical but has been observed across numerous African countries. Case studies from Cameroon’s Bamendjin Dam,^14^ Kenya’s Kamburu Dam,^15^ Ethiopia’s Gilgel Gibe Dam^16^ and Ghana’s Akosombo Dam^17^ highlight a recurring pattern: increased proximity to large dam reservoirs corresponds to heightened malaria prevalence.

Western and SSA, in particular, bear a disproportionately high malaria burden, with *Plasmodium falciparum* (the deadliest malaria parasite) accounting for the vast majority of cases, with children under five and pregnant women facing the greatest risks.^18^ Across the African continent in 2022, although there was an estimated 249 million malaria cases and 608 000 malaria deaths, the SSA region carried a disproportionately higher share of global rates, home to 94% of malaria cases (233m) and 95% (580 000) of malaria-related deaths^18^ where a child reportedly dies every 2 min.^19^ An influential study by Kibret et al. reported that between 2000 and 2015, the number of people living within 5 km of large dam reservoirs in SSA grew by around 4.3m – rising from 14.4m to 18.7m.^20^ As such, a rapid increase in dam construction across malaria-endemic regions such as Western and SSA has raised important questions about the potential ecological and epidemiological consequences of such infrastructure projects.

## Research methods and design

### Study setting

**FIGURE 1:**
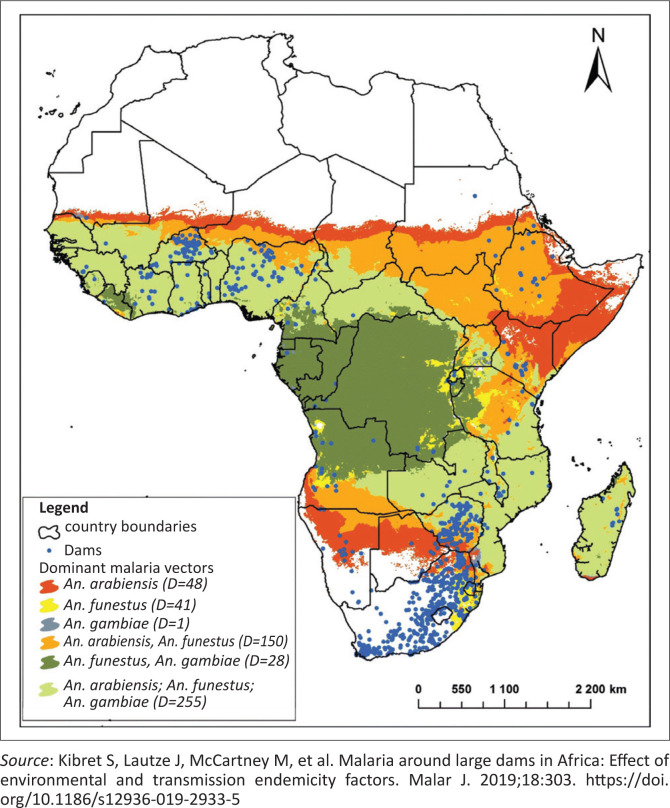
Distribution of dams in relation to dominant malaria vectors across sub-Saharan Africa.

In Western Africa, the primary vectors surrounding large dams are *Anopheles arabiensis, Anopheles funestus* and *Anopheles gambiae*. Understanding the spatial relationship between these vectors and dam locations is critical to evaluating whether these projects may exacerbate malaria risk. Figure 1^10^ presents a spatial overview of the geographic distribution of major dams (indicated by blue dots) overlaid with dominant malaria vector zones across SSA. The map categorises regions by the prevalence of the three key malaria vectors using colour-coded shading, with darker green areas representing regions where all three vectors are simultaneously dominant. The map highlights that *A. arabiensis* is most prevalent near dams situated in ecologically unstable or semi-arid areas, while zones of high vector diversity – marked by the presence of all three vectors – are located in more humid, stable transmission zones.

### Côte d’Ivoire’s malaria epidemiology profile

*Plasmodium falciparum* dominates the malaria landscape in Côte d’Ivoire, comprising 98% – 99% of all cases. Within the country, 58 out of its 113 districts grapple with high endemicity, registering 300–499 cases per 1000 individuals.^21^ Notably, the national annual incidence rate stands at 441 cases per 1000 among children below 5 years old and 173 cases per 1000 across the general populace.^21^ Côte d’Ivoire ranks among the top 10 nations globally with the highest malaria burdens. In 2020 alone, it contributed to 3.0% of global cases and deaths, representing 2.4% of worldwide fatalities and 6% of malaria instances in West Africa.^21^

### Gabon’s malaria epidemiology profile

Malaria is highly prevalent in Gabon, where transmission occurs year-round.^22^ The disease burden follows a seasonal pattern influenced by the equatorial climate, which fosters the proliferation of mosquitoes and larval development.^23^ Gabon reports a high malaria incidence rate estimated at 97.5 cases per 1000 individuals.^22^ In Gabon, malaria ranks as the second leading cause of consultation and hospitalisation in paediatric wards, following respiratory tract infections. It affects over one-third of all febrile patients, with almost all cases attributed to *P. falciparum*, exhibiting prevalence rates ranging from 94% to 99%.^24^

### Ghana’s malaria epidemiology profile

Malaria is endemic in Ghana, with transmission persisting throughout the year, especially in the northern regions, which experience distinct seasonal variations. The southern and middle parts of the country observe two pronounced rainy seasons from April to June and September to November. Comprising 2.2% of global malaria cases and deaths, with 2% of worldwide malaria fatalities, Ghana ranks among the 15 countries worldwide with the highest malaria burdens.^25^ Additionally, it contributes to 4% of malaria cases in West Africa.^25^ Notably, Ghana exhibited significant progress in malaria control between 2020 and 2021, maintaining stable case rates at 165 cases per 1000 of the population at risk.^26^ Although there was a slight decrease of 1.7% in deaths during the same period (decreasing from 0.39 to 0.38 per 1000 of the population at risk), malaria remains a consistent public health challenge for the country.^25^

A summary of the three implemented hydrodam projects examined in this study can be found in Table 1, including the Soubré Dam in Côte d’Ivoire, the Grand Poubara Dam in Gabon and the Bui Dam in Ghana. Each row provides essential contextual details necessary for temporal and geographic alignment of the Difference-in-Differences (DiD) analysis, including the project’s financier (China EXIM Bank), the construction company (Sinohydro), geographic location (by region or province), installed energy capacity (in megawatts) and construction timeline. Critically, the final column outlines the relevant United States Agency for International Development (USAID) Demographic and Health Survey (DHS) datasets used to capture pre- and post-construction health and socio-economic data for each project area. This alignment enables a consistent baseline and post-intervention comparison period across all three cases.

**TABLE 1:** Overview of assessed hydropower projects details.

Country	Financier	Contract builder	Hydrodam project	Province	Megawatts produced	Construction timeline	DHS dataset used
Côte d’Ivoire	China EXIM bank	Sino hydro	Soubré hydrodam	Bas-Sassandra region	275 MW	2013–2017	2010–2020
Gabon	China EXIM bank	Sino hydro	Grand Poubara hydrodam	Haut-Ogooué Province	160 MW	2008–2014	2005–2015
Ghana	China EXIM bank	Sino hydro	Bui hydrodam	Savannah region	400 MW	2007–2013	2005–2015

DHS, Demographic and Health Survey.

### Data description

The data utilised in this study originate from the DHS database managed by the USAID.^27^ The DHS programme is a large-scale survey initiative that collects data on population, health, and socio-economic indicators across developing countries. These surveys are typically conducted in collaboration with national statistical agencies and international organisations.

The study incorporates geospatial covariate data from the DHS database to control for potential confounding variables when analysing the causal relationship under investigation. The specific DHS cluster variables included in this analysis, along with their measurement descriptions, are outlined further in the text:

- **Malaria incidence:** Number of clinical cases of *Plasmodium falciparum* malaria per person.
- **Malaria prevalence:** Parasite rate of *Plasmodium falciparum* in children between the ages of 2 years and 10 years old.
- **Elevation:** Shuttle Radar Topography Mission (SRTM) near-global Digital Elevation Models (DEMs) in metres.
- **Global human footprint:** Global Human Footprint index (from extremely rural 0 to extremely urban 100).
- **Irrigation:** Proportion of area equipped for irrigation.
- **Insecticide-treated bed nets (ITN) coverage:** Proportion of the population protected by ITNs.
- **Mean temperature:** Mean temperature.
- **Rainfall:** Annual rainfall (per year).

### Data analysis

The study employs Geographic Information System (GIS) analysis to visualise and interpret spatial data. The first step involved mapping the DHS sub-boundary data for Côte d’Ivoire, Gabon and Ghana using spatial shapefiles representing administrative boundaries. This mapping process was conducted using the ‘leaflet’ package in R to create an interactive web-based map of the study areas. Subsequently, the DHS geospatial covariate data, which represent the locations of survey clusters, were overlaid onto the web-map. The hydrodam locations were then integrated using their respective geographic coordinates. A 15-km radius was constructed around each hydrodam site using the ‘geom’ package, establishing the treatment zone (DHS cluster households falling within this radius). All remaining DHS clusters within the administrative region were designated as the control group.

**FIGURE 2:**
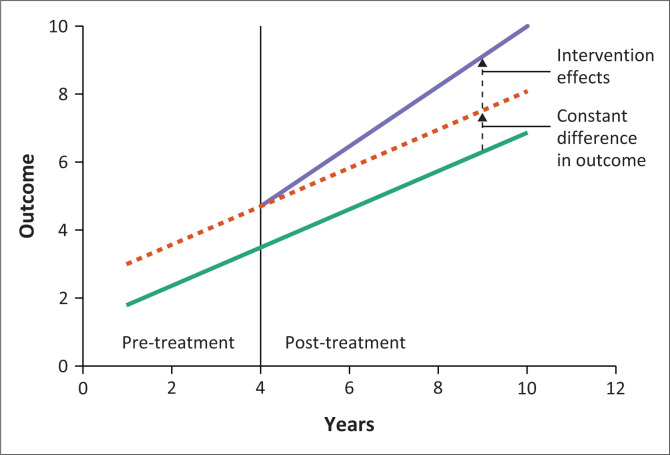
Conceptual framework of a difference-in-difference analysis.

A DiD approach was applied to estimate the causal effect of the intervention, as seen in Figure 2 which provides a conceptual framework of DiD analysis. This method compares changes in outcomes over time between a treatment group (households within 15 km of a hydrodam) and a control group (households outside this radius but within the same administrative region).

The DiD method relies on comparing outcome trends before and after an intervention between treated and control groups. To estimate causal effects reliably, this requires data across multiple time periods – both pre-intervention (to establish baseline trends) and post-intervention (to capture the treatment effect over time). As shown in Table 2, the analysis incorporates multiple time periods before and after hydrodam construction to assess the impact of the intervention. The table outlines the alignment between hydrodam construction timelines and the corresponding DHS datasets used in the analysis across the three countries. This includes both the range of DHS survey data (covering multiple years pre- and post-construction) and the construction period for each dam, as well as the average temporal spread (also referred to as ‘average variance’) between the two. For example, Ghana’s DHS data span a period of 2005–2015, covering multiple years both before and after the Bui hydrodam construction (2008–2014). This temporal spread ensures that the study provides adequate coverage of pre-treatment trends and post-treatment effects, enabling a more robust estimation of the causal impact of hydrodam implementation on malaria incidence and prevalence.

**TABLE 2:** Comparison of assessed hydropower project construction timelines and DHS data periods.

Country	Hydrodam	DHS dataset used	Construction timeline	Average variance (years)
Côte d’Ivoire	Soubré hydrodam	2010–2020	2013–2017	3
Gabon	Grand Poubara hydrodam	2005–2015	2008–2014	2
Ghana	Bui hydrodam	2005–2015	2008–2014	2

DHS, Demographic and Health Survey

### Difference-in-difference regression model

Malaria Incidence Model (see Equation 1):

$$\begin{array}{l} Malaria_{I}=\beta_{0}+\beta_{1}\;Treatment+\beta_{2}\;Post+\beta_{3}\;Treatment\times Post+\beta_{4}\\ Elevation+\beta_{5}\;Irrigation+\beta_{6}\;GlobalHumanFootpr\mathrm{int}+\beta_{7}\\ INTCoverage+\beta_{8}\;MeanTemperature+\beta_{9}\;Rainfall+\varepsilon \end{array}$$ [Eqn 1]

Malaria Prevalence Model (see Equation 2):

$$\begin{array}{l} Malaria_{p}=\beta_{0}+\beta_{1}\;Treatment+\beta_{2}\;Post+\beta_{3}\;Treatment\times Post+\beta_{4}\\ Elevation+\beta_{5}\;Irrigation+\beta_{6}\;GlobalHumanFootpr\mathrm{int}+\beta_{7}\\ INTCoverage+\beta_{8}\;MeanTemperature+\beta_{9}\;Rainfall+\varepsilon \end{array}$$ [Eqn 2]

where:

- *Malaria_incidence* is the dependent variable representing the incidence of malaria.
- *Malaria_prevalence* is the dependent variable representing the prevalence of malaria.
- *Treatment* is a binary indicator variable taking the value 1 if the observation is in the treatment group and 0 otherwise.
- *Post* is a binary indicator variable taking the value 1 if the observation is in the post-treatment period and 0 otherwise.
- *Treatment × Post* is the interaction term between treatment and post-treatment period, which captures the treatment effect. This is the DiD estimator – which captures the difference in change between the dependent variables (malaria prevalence and incidence rates), between the treatment (DHS cluster households within a 15 km radius) and control (DHS cluster households within the administrative region) groups after the intervention (the implementation of the Hydrodam Project).
- *Control variables* represent additional control variables included in the model, such as elevation, irrigation, global human footprint, ITN coverage, mean temperature and rainfall.
- *β0, β1, β2, β3, β4, β5, β6, β7, β08, β9, β10* are the coefficients associated with each independent variable in the model, representing the estimated effect of each variable on malaria incidence, holding other variables constant.
- *ϵ* represents the error term, capturing unobserved factors or random variation that may affect malaria incidence but are not accounted for in the model.

## Results

Table 3 presents the Difference-in-Difference regression results on malaria incidence and prevalence across Côte d’Ivoire, Gabon and Ghana between the interventions effects, showing the impact of the treatment, along with key environmental and socio-economic control variable factors.

**TABLE 3:** Difference-in-differences regression results for malaria incidence and prevalence in assessed hydropower project areas (15 km radius).

Country	Variable	Malaria incidence				Malaria prevalence			
		Estimate	*Pr*(>|*t*|)	Statistical significance	Adjusted *R*-squared	Estimate	*Pr*(>|*t*|)	Statistical significance	Adjusted *R*-squared
Côte d’Ivoire	Treatment	0.0002327	0.989706	-	0.9283	−0.01364	0.67689	-	0.9364
Côte d’Ivoire	Post	−0.3165	0.000233	***	-	−0.3098	0.040242	*	-
Côte d’Ivoire	Treatment Post	0.03051	0.210263	-	-	0.0566	0.200177	-	-
Côte d’Ivoire	Elevation	0.0004587	0.00000495	***	-	0.0006452	0.000275	***	-
Côte d’Ivoire	Irrigation	0.001145	0.844565	-	-	0.006339	0.549972	-	-
Côte d’Ivoire	Global Human Footprint	−0.001922	0.000942	***	-	−0.003879	0.000262	***	-
Côte d’Ivoire	ITN Coverage	0.3346	0.039808	*	-	−0.3681	0.20829	-	-
Côte d’Ivoire	Mean Temperature	0.04635	0.088094	-	-	0.09376	0.057669	-	-
Côte d’Ivoire	Rainfall	−0.000002793	0.778227	-	-	0.00001773	0.325703	-	-
Gabon	Treatment	−0.0132171	0.562284	-	0.6157	−0.0132139	0.651503	-	0.5756
Gabon	Post	−0.126114	0.056234	-	-	−0.1042753	0.214365		-
Gabon	Treatment Post	−0.0003839	0.990292	-	-	−0.0004847	0.990449	-	-
Gabon	Elevation	0.0002951	0.015216	*	-	0.0003018	0.050894	-	-
Gabon	Irrigation	−0.0006461	0.805846	-	-	−0.0010361	0.758798	-	-
Gabon	Global Human Footprint	−0.0022535	0.000127	***	-	−0.0024834	0.000825	***	-
Gabon	ITN Coverage	0.8590207	0.034486	*	-	0.7545992	0.143634	-	-
Gabon	Mean Temperature	0.0134183	0.604414	-	-	0.0116545	0.725703	-	-
Gabon	Rainfall	−0.000369	0.012585	*	-	−0.0004061	0.03113	*	-
Ghana	Treatment	0.01476	0.6403	-	0.8141	0.02213	0.683914	-	0.8232
Ghana	Post	−0.2586	2.25E-09	***	-	−0.4038	2.96E-08	***	-
Ghana	Treatment Post	−0.0761	0.08889	-	-	−0.1256	0.10228	-	-
Ghana	Elevation	0.0001264	0.22208	-	-	0.00006277	0.723128	-	-
Ghana	Irrigation	−0.03358	0.03323	*	-	0.00006277	0.018925	*	-
Ghana	Global Human Footprint	−0.0004848	0.4847	-	-	−0.001065	0.372789	-	-
Ghana	ITN Coverage	0.3637	0.00000377	***	-	0.5144	0.0000961	***	-
Ghana	Mean Temperature	−0.04525	0.03034	*	-	−0.121	0.001028	**	-
Ghana	Rainfall	0.000201	0.00176	**	-	0.0003467	0.001723	**	-

Note: In difference-in-differences (DiD) regression analysis, asterisks (*) indicate the statistical significance of estimated coefficients, showing whether an observed effect is unlikely to have occurred by chance.

ITN, insecticide-treated bed nets.

* , Typically denotes significance at the 10% level;

** , at 5%;

*** , at 1%, meaning that coefficients with more asterisks are typically more robustly statistically significant.

### Strength of the models: Adjusted *R*-squared values

As seen in Table 3, one of the first observations from the DiD analysis is the consistently high adjusted *R*-squared values across the models for Côte d’Ivoire, Gabon and Ghana, indicating that the selected independent variables explain a significant proportion of the variation in malaria prevalence and incidence.

In Côte d’Ivoire, the model demonstrates a strong fit, with an adjusted *R*-squared of 0.9283 for malaria incidence and 0.9364 for malaria prevalence. Gabon also exhibits relatively high explanatory power, with an adjusted *R*-squared of 0.6157 for malaria incidence and 0.5756 for malaria prevalence. Similarly, in Ghana, the model achieves strong explanatory power, with an adjusted *R*-squared of 0.8141 for malaria incidence and 0.8232 for malaria prevalence. These high *R*-squared values underscore the robustness of the models and the relevance of the chosen predictors in understanding malaria epidemiology across the three countries.

### The difference-in-differences estimator

The *Treatment Post* variable, the DiD estimator, does not show a statistically significant increase in malaria incidence or prevalence following hydrodam construction across Côte d’Ivoire, Gabon and Ghana. In Côte d’Ivoire, the effect on incidence (*p* = 0.210) and prevalence (*p* = 0.200) is not statistically significant. Similarly, in Gabon, neither malaria incidence (*p* = 0.990) nor prevalence (*p* = 0.990) exhibits significant changes. In Ghana, the effect on malaria incidence (*p* = 0.089) and prevalence (*p* = 0.102) is also not statistically significant. Thus, despite the expectation that new reservoirs might create breeding sites, the analysis did not find a consistent, significant rise in malaria following dam construction in these settings.

### Global human footprint

The Global Human Footprint variable consistently emerges as a significant negative predictor of malaria prevalence and incidence across the three countries. In Côte d’Ivoire, malaria incidence decreases significantly with an increase in human footprint (*β* = –0.001922, *p* = 0.000942), and malaria prevalence follows a similar trend (*β* = –0.003879, *p* = 0.000262). In Gabon, the impact is even stronger, with malaria incidence (*β* = –0.0022535, *p* = 0.000127) and prevalence (*β* = –0.0024834, *p* = 0.000825) both showing highly significant negative correlations. These findings reinforce the view that areas with higher levels of human modification of the environment tend to experience reduced malaria transmission.

### Insecticide-treated bed net coverage

In several models, ITN coverage is statistically significant, but its association with malaria prevalence and incidence appears paradoxical at first. Instead of reducing malaria rates, ITN coverage is positively correlated with increased incidence in some cases. In Côte d’Ivoire, ITN coverage is positively associated with malaria incidence (*β* = 0.3346, *p* = 0.0398), indicating that areas with higher malaria risk tend to receive more ITNs. In Ghana, the relationship is even more pronounced, with ITN coverage showing a strong positive correlation with malaria incidence (*β* = 0.3637, *p* = 0.00000377) and prevalence (*β* = 0.5144, *p* = 0.0000961). In Gabon, the relationship is also positive but less statistically robust (*β* = 0.859, *p* = 0.0345 for incidence and *p* = 0.1436 for prevalence). This seemingly counterintuitive result likely reflects the fact that ITNs are distributed primarily in high-risk areas where malaria transmission is already prevalent. The positive correlation does not necessarily indicate that ITNs cause an increase in malaria but rather that their distribution is a response to high transmission risk.

### Elevation

Elevation plays a significant role in malaria prevalence and incidence across all three countries. In Côte d’Ivoire, elevation is positively correlated with both malaria incidence (*β* = 0.0004587, *p* = 0.00000495) and prevalence (*β* = 0.0006452, *p* = 0.000275), meaning higher-altitude areas are seeing increased malaria cases. In Gabon, the effect is also positive and statistically significant, though slightly weaker (*β* = 0.0002951, *p* = 0.0152 for incidence, and *β* = 0.0003018, *p* = 0.0509 for prevalence). These results indicate increasing cases at higher altitudes in these countries, especially in Côte d’Ivoire and Ghana.

### Rainfall

Rainfall’s relationship with malaria transmission varies. In Gabon, rainfall is negatively correlated with malaria incidence (*β* = –0.000369, *p* = 0.0126) and prevalence (*β* = –0.0004061, *p* = 0.0311), meaning that while rainfall contributes to mosquito breeding, excessive rainfall might be washing away breeding sites. However, in Ghana, rainfall is highly significant at the 1% level for both incidence (*β* = 0.000201, *p* = 0.00176) and prevalence (*β* = 0.0003467, *p* = 0.001723), suggesting a direct and strong impact on malaria transmission. In Côte d’Ivoire, rainfall was not significant in the models.

### Temperature

Mean temperature is a significant predictor of malaria prevalence and incidence, though its effects vary by country. In Ghana, a higher mean temperature is associated with reduced malaria prevalence, as indicated by the negative coefficient (*β* = –0.121) and strong statistical significance (*p* = 0.001028). A similar negative but less significant relationship is observed in malaria incidence (*β* = –0.04525, *p* = 0.03034). These findings imply that higher temperatures may reduce mosquito survival and parasite development, potentially limiting malaria transmission.

Interestingly, Côte d’Ivoire does not exhibit the same pattern. In Côte d’Ivoire, temperature has a positive but weakly significant relationship with malaria prevalence (*β* = 0.09376, *p* = 0.057669) and malaria incidence (*β* = 0.04635, *p* = 0.088094). In Gabon, temperature does not significantly impact malaria prevalence or incidence.

### Irrigation

The impact of irrigation on malaria prevalence and incidence appears to be highly context-dependent, as evidenced by its varying statistical significance across countries. In Ghana, irrigation is a significant factor, with a negative coefficient (*β* = –0.03358, *p* = 0.03323), suggesting that increased irrigation is associated with reduced malaria incidence. A similar negative relationship is observed for malaria prevalence (*β* = –0.018925, *p* = 0.018925). This could indicate that well-managed irrigation systems, possibly with proper drainage or biological control measures (e.g. fish that eat mosquito larvae), mitigate malaria risks. However, in Côte d’Ivoire and Gabon, irrigation does not emerge as a significant factor.

## Discussion

### Interpretation of the results

The models indicate that environmental and socio-economic factors including Global Human Footprint, ITN coverage and elevation are the strongest predictors in explaining malaria well (high *R*^2^), but the addition of large hydroelectric dams alone did not produce a uniform increase in malaria. The non-significant ‘Treatment_Post’ (the DiD estimator) coefficients suggest that new reservoirs did not, on average, lead to detectable spikes in malaria incidence or prevalence in the studied provinces.

The consistently negative effect of human footprint (urbanisation) on malaria is notable. This aligns with the urban malaria theory: developed areas typically have better housing, drainage and health infrastructure, which reduce malaria.^28^ In line with this, the results show that provinces with greater infrastructure development tend to have lower malaria rates. However, while urbanisation reduces malaria incidence overall, it is important to note that localised pockets of transmission can persist in rapidly growing peri-urban areas where inadequate sanitation and drainage systems allow mosquito breeding.

The positive association of ITN coverage with malaria likely reflects reactive distribution. Guidelines recommend targeting nets to highest risk communities.^29^ Thus, areas with severe malaria problems receive more ITNs, producing a positive statistical correlation. This does not imply that nets increase malaria, but rather that high-coverage areas are those suffering greater burden (i.e. an indication of targeting efficacy).

The elevation effect – higher malaria at higher altitude – may seem counterintuitive as cooler highlands usually have less transmission. However, it is increasingly documented that African highlands are experiencing malaria expansion, possibly because of climate change and local land-use changes.^30^ The finding of significant elevation effects in Côte d’Ivoire and Gabon echoes studies in East Africa that have observed malaria moving upslope.^31,32,33^ These highland populations are often non-immune, so even small environmental shifts can raise local transmission.

In terms of environmental predictors, such as rainfall, temperature and irrigation, the results also fit known patterns. Moderate rain creates breeding pools, but extremely heavy rain can wash larvae away.^34^ This explains why Gabon (very wet) shows a negative rainfall coefficient, whereas Ghana (monsoonal rains) shows the expected positive association. Temperature results reflect the non-linear biology of malaria: transmission peaks at moderate temperatures but declines if it becomes too hot for mosquito survival or parasite development.^35^ Finally, irrigation’s effect in Ghana is supported by literature that well-managed irrigation (with proper drainage or larvivorous fish) can actually reduce malaria incidence.^36^

### Public health and policy implications

These results have several implications for malaria control around hydropower projects. Reservoir management should consider malaria: for example, manipulating water levels to avoid prolonged shallow pools after heavy rain can disrupt breeding. The rainfall findings suggest scheduling reservoir drawdowns to prevent stagnant water pools in the critical weeks post-inundation.

Environmental design also matters. The positive elevation effect implies that steeper dam slopes and stabilised shorelines (e.g. with native vegetation or predatory fish) could reduce shoreline pools that form at higher water lines. Engineering dams with mosquito control in mind can also mitigate transmission risk.^37^

Urban development near dams provides an opportunity: as the models show that urbanisation correlates with lower malaria, integrating infrastructure improvements (like drainage, housing, health clinics) into dam-area planning could amplify this effect.^38,39^ For example, building proper sewage and water drainage in new dam towns can exploit the protective urban footprint effect observed in the study.

Traditional vector control programmes must still play an important role. High ITN coverage in dam regions is a good sign of targeting, but nets alone are insufficient.^40^ Continuous community outreach (e.g. indoor spraying, larval source management, health education) should accompany net distribution. Real-time surveillance, including entomological monitoring around reservoirs, can help detect any emergent hotspots. Climate-based early-warning systems might leverage the findings on temperature and rainfall to time interventions. Policymakers and developers working on hydropower projects should therefore integrate malaria mitigation (as we have recommended) into project planning and community development.

### Research limitations

This study provides valuable insights into the malaria transmission impacts of implemented hydrodam projects across Western Africa; however, several limitations should be acknowledged.

Establishing a direct causal relationship between hydrodam implementation and malaria outcomes remains challenging, as unobserved factors such as local health infrastructure, population mobility and land-use changes may influence malaria transmission despite the use of DiD regression modelling. The 10-year pre- and post-construction observation period may not fully capture longer-term climate-driven shifts in malaria risk, highlighting the need for future longitudinal studies incorporating climate projections.

The reliance on province-level geospatial data limits the ability to detect localised variations in malaria transmission linked to socio-economic disparities, while inconsistencies in DHS cluster representation reduce the robustness of findings. Additionally, the study does not explicitly account for seasonal malaria transmission patterns, which could provide further insights into the effectiveness of climate-responsive interventions. Expanding the dataset to include higher-frequency health data, finer-scale geospatial mapping and extended timeframes would strengthen causal inferences and enhance targeted public health interventions.

## Conclusion

This study examines the relationship between hydrodam implementation and malaria outcomes in Western Africa, using DiD regression modelling and GIS analysis to assess the impact of Sinohydro hydrodam projects in Côte d’Ivoire, Gabon and Ghana. While initial observations suggested a rise in malaria prevalence post-construction, statistical analysis found no significant causal relationship, instead highlighting elevation, human footprint, rainfall and temperature as key predictors of malaria incidence. These findings underscore the need for proactive malaria mitigation strategies in hydrodam-affected regions. Policy recommendations include climate-responsive malaria control programmes, reservoir water level management, strengthened health impact assessments and community-based prevention initiatives. By integrating these measures into hydrodam planning, policymakers can enhance both energy security and public health outcomes, ensuring more sustainable infrastructure development in Africa.
